# No Elevated Plasma Catecholamine Levels during Sleep in Newly Diagnosed, Untreated Hypertensives

**DOI:** 10.1371/journal.pone.0021292

**Published:** 2011-06-17

**Authors:** Björn Rasch, Christoph Dodt, Friedhelm Sayk, Matthias Mölle, Jan Born

**Affiliations:** 1 Department of Neuroendocrinology, University of Lübeck, Lübeck, Germany; 2 Division of Biopsychology, University of Zürich, Zürich, Switzerland; 3 Department of Internal Medicine, University of Lübeck, Lübeck, Germany; 4 Division of Emergency Medicine, München-Bogenhausen Hospital, München, Germany; 5 Department of Medical Psychology and Neurobiology, University of Tübingen, Tübingen, Germany; University Institute of Social and Preventive Medicine, Switzerland

## Abstract

The sympatho-adrenergic system is highly involved in regulating sleep, wake and arousal states, and abnormalities in this system are regarded as a key factor in the development and progression of arterial hypertension. While hypertension is associated with a hyperadrenergic state during wakefulness, the effect of hypertension on plasma-catecholamine levels during sleep is not yet known. Twelve young participants with newly diagnosed, untreated hypertension and twelve healthy controls slept for 7 hours in the sleep laboratory. Before and after sleep, subjects rested in a supine position for 3-h periods of wakefulness. We sampled blood at a fast rate (1/10 min) and monitored blood pressure and heart rate continuously. We show that plasma NE and E levels did not differ between hypertensives and normotensive during sleep as well as before and after sleep. Blood pressure was higher in hypertensives, reaching the largest group difference in the morning after sleep. Unlike in the normotensives, in the hypertensive participants the morning rise in blood pressure did not correlate with the rise in catecholamine levels at awakening. Our results suggest that hypertension in its early stages is not associated with a strong hyperadrenergic state during sleep. In showing a diminished control of blood pressure through sympatho-adrenergic signals in hypertensive participants, our data point towards a possible involvement of dysfunctional sleep-related blood pressure regulation in the development of hypertension.

## Introduction

The sympathetic system exerts a key role in homeostatic blood pressure control. It has been hypothesized that an abnormality in sympathetic control of the cardiovascular system participates in the development and progression of hypertension ([1], for a review). This “neuroadrenergic hypothesis of hypertension” was established in patients suffering either from secondary end-organ damage of heart and kidneys or from comorbidities that induce sympatho-excitation like the metabolic syndrome or sleep-related breathing disorders. Indeed, also several studies have provided evidence that even in young hypertensives plasma levels of norepinephrine (NE) as well as other markers of sympathetic drive, are increased [1]–[4]. However, whereas these studies were conducted during active wakefulness, the effect of hypertension on plasma NE levels during nocturnal sleep is still unknown.

Recent studies have renewed interest in the importance of sleep-related sympathetic nervous processes for the pathophysiology of hypertension. Normal nocturnal sleep is characterized by a marked decrease in sympathetic activity and blood pressure, with these processes also affecting sympathetic regulation of blood pressure during subsequent daytime wakefulness [5]. Moreover, there is growing evidence pointing to a significant contribution of sleep and sleep-related control of blood pressure to the development of hypertension [6]–[8]. In addition, epidemiological studies have consistently associated short sleep duration and poor sleep quality with higher blood pressure, and these sleep parameters readily predicted increases in blood pressure over the next 5 years [9], [10]. Conversely, chronic sleep disturbances in the case of sleep apnea-hyponea are strongly linked to an increased risk of hypertension [11] and experimental sleep deprivation increases blood pressure in both animals and humans (see [12], for a review). However, the underlying processes remain to be elucidated. One important factor might be related to alterations of the sympatho-adrenergic activity during sleep.

Therefore, in the present study, we investigated whether hypertension alters catecholaminergic activity during sleep. Based on the neuroadrenergic hypothesis of hypertension [1], we predicted that sympatho-adrenergic activity should be elevated during sleep already in early hypertension. To avoid confounding effects of variables like obesity, sleep fragmentation and sleep apnea-hyponea, we only included young, otherwise healthy participants with newly diagnosed, untreated primary hypertension in our experimental group. Twelve normotensive and the twelve young hypertensive participants slept a regular 7-h period of nocturnal sleep in the sleep laboratory. Subjects remained in a horizontal position for 3-h periods of wakefulness before and after sleep. Blood pressure and heart rate were recorded continuously, and plasma catecholamines were sampled repeatedly at a fast rate (every 10 min) in order to correlate sympathoadrenergic activity with sleep stages. The design allowed us to investigate whether hypertension affected plasma levels of NE and E during sleep compared to wakefulness before and after sleep as well as depending on the sleep stage (NonREM vs. REM).

## Methods

### Subjects

12 normotensive men (26.4±1.7 years) and 12 men with newly diagnosed untreated primary hypertension (26.3±3.5 years) participated in the experiments. All participants were students at the University of Lübeck and were paid for participation. Subjects were healthy, non-smoking, did not report any sleep disturbances, and had not worked on night-shift for at least 8 weeks. The study was approved by the local ethics committee and all participants gave written informed consent.

Student subjects were recruited during a 6-months period on the campus of the University of Lübeck (by advertising in the students' cafeteria, lectures etc.). Particularly those with a positive family history of hypertension were addressed in the advertisement. Altogether 180 male students were evaluated over a period of 4 weeks before 12 hypertensive subjects and 12 controls were found. The normotensive control subjects were recruited from the same group. A comprehensive medical history, and physical examination and a laboratory screening (creatinine, electrolytes, urinalysis) confirmed that all included subjects were otherwise healthy. Additionally, all subjects were non-smoking, did not report any sleep disturbances, and had not worked on night-shift for at least 8 weeks prior to participation. The study was approved by the local ethics committee and all participants gave written informed consent.

The hypertensive men were selected on the basis of repeated oscillometric blood pressure measurements (Welsh Allyn®). This “Vital signs” monitor has been validated previously [13]. The inclusion criterion was a systolic blood pressure higher than 140 mmHg and a diastolic blood pressure higher than 90 mmHg on three subsequent measurements (each measurement at least 2 min apart) obtained while the subject was sitting after a 10 min period of rest. Hypertensive and normotensive subjects were matched regarding age and body mass index (see Table 1 for anthropometric data). There were no differences between the normotensive and early hypertensive groups in body mass index (22.86±6.6 versus 23.95±9.7, *P*>0.75), reported daily physical activity and sleep parameters on the adaptation nights (all *P*>0.3). In the pre-experimental interviews subjects of the early hypertensive group expressed slightly more often hints towards a family history of hypertension. Data of normotensive participants were partly published elsewhere [14].

**Table 1:** Subjects' characteristics.

	Normotensives	Hypertensives
Age (yrs)	26.4±5.9	26.3±12.1
Body mass index (kg/m^2^)	22.86±6.6	23.95±9.7
Systolic blood pressure (mmHg)	125.1±27.7	156.9±50.9*
Diastolic blood pressure (mmHg)	72.6±25.3	90.6±29.8*

Age, body mass index, diastolic and systolic blood pressure of the 12 normotensive and 12 hypertensive subjects (means ± standard deviation). Blood pressure values are means of three consecutive oscillometric measurements in sitting subjects after a rest of 10 min. *p<0.05 compared to the respective blood pressure values of normotensives.

### Procedure

Subjects were adjusted to the experimental setting by spending at least one prior night under conditions of the experiment including standard polysomnographic recordings and the placement of catheters for blood collection. The men were also adjusted to continuous blood pressure recording measured photoplethysmographically at the finger using the Finapres technique (Ohmeda Monitoring Systems, Englewood, CO; [15]). While this method allows continuous non-invasive blood pressure monitoring during prolonged intervals, absolute values of blood pressure may differ from those obtained by oscillometric measurements. The measurement required the fixation of the arm and finger used to attach the pressure cuff, and in turn a relatively constant sleeping position either on the back or front. For adapting the subject to this procedure, during two nights at home and during an additional night at the sleep laboratory (preceding the adaptation night) he slept in the same body position as during the experiments with one arm fixated to the bed frame. Although habituation to arm fixation may not have been completed after the fourth night, sleep recordings on the experimental night as well as interviews with the subjects did not reveal any hint at a substantial sleep disturbance introduced by this procedure. For these adaption nights, it was carefully controlled that there were no increases in signs of sleep fragmentation, arousals or apnoeas during sleep, and there were also no differences in these signs between the experimental groups, i.e., all participants exhibited healthy normal sleep without any signs of increased arousals or apnoea. Although this is a limiting factor of our study, we did not directly monitor blood oxygen levels to avoid additional disturbing effects on sleep in the laboratory, and because we did not detect any hints for the presence of sleep apneas in any of our subjects based on the pre-experimental interviews and the recordings in the adaptation nights.

During the wake periods preceding experimental sleep periods, subjects had to abstain from coffee, black tea and alcoholic drinks, but to maintain their normal diet. They had eaten a regular meal 2 h before recordings started. They were not allowed to take any naps during the wake periods preceding experimental epochs. Subjects arrived at the laboratory ∼1 h before the start of recordings, to prepare standard polysomnography, blood sampling and recordings of blood pressure.

Recordings started at 20:00 h. After a 30-min phase of habituation, subjects spent awake for the 3-h interval between 20:30 and 23:30 h. Then, lights were turned off to enable sleep for 7 h, until 06:30 h, when they were gently awoken. Subjects stayed awake for another 3-h period between 06:30 and 09:30 h. During the periods of wakefulness, they were allowed to read magazines or to talk to the experimenters. Subjects remained in a supine position throughout the entire recording epoch between 20:00 and 09:30 h.

Standard polysomnographic recordings [16] included continuous monitoring of electroencephalogram, electrooculogram and electromyogram. For blood sampling, a catheter was inserted into a forearm vein and connected to a long thin plastic tube that enabled blood collection from an adjacent room without disturbing the subject's sleep. Blood was sampled every 10 min throughout the recording epochs. To prevent clotting and to substitute removed blood volume saline solution was infused slowly throughout the recording epoch totaling a volume of ∼300 ml.

### Data Reduction and Statistical Analyses

Polysomnographic recordings were scored offline, independently by two experienced experimenters, according to the standard criteria [16]. Subsequent 30-s epochs of recordings were scored visually as intermittent wakefulness (I-Wake), Non-REM sleep stages 1, 2 (S1–S2), or slow-wave-sleep (SWS, defined by the sum of S3–4), and REM sleep. For each night, total sleep time (in min) and the percentage of total sleep time spent in the different sleep stages were determined. Sleep onset latency was defined by the first epoch of S1 sleep followed by S2 sleep and determined with reference to the time of lights off. Latency of S2 sleep, SWS, and REM sleep was calculated with reference to sleep onset.

For determination of plasma concentrations of NE and E, blood samples were centrifuged immediately after collection, and plasma was stored at −80°C until assay. NE and E were determined by standard high performance liquid chromatography with subsequent electrochemical detection (Chromsystems Instruments and Chemicals, Munich Germany [17]). The sensitivity was 6.48 pg/ml for E and 6.02 pg/ml for NE. The interassay coefficients of variation were 5.6% and 6.1% for E and NE, respectively. Intraassay coefficients of variation were confirmed by measuring a small fraction of sample from this study in duplicate in the same assay. Because blood was sampled every 10 minutes during the entire experimental procedure (total duration approx. 13 h), approximately 78 samples were obtained for each individual, providing altogether a reliable basis for hormonal measurements.

Average values were calculated of plasma NE and E concentrations, and of heart rate, systolic and diastolic blood pressure for the 3-hour wake periods before (‘pre-wake’ period) and after sleep (‘post-wake’ period) and for the time in each sleep stage. Calculations were based on plasma concentrations of NE and E for subsequent 30-s intervals as determined by linear interpolation.

Effects were statistically evaluated using analyses of variance (ANOVA) including the between-subject factors ‚group' (hypertensives vs. normotensives) and the repeated measures factor ‘sleep/wake’ periods (pre-wake, sleep, post-wake) or for ‘sleep stage’ (intermittent wake time, S1, S2, SWS, REM sleep). No data from any variable significantly deviated from the normal distribution as indicated by Kolomogorov-Smirnov Tests. To account for violations of the sphericity-assumption, degrees of freedom were adjusted using the Greenhouse-Geisser procedure in SPSS 16.0. Significant main effects of sleep/wake or sleep stage were specified by planned pair-wise contrasts with reference to sleep or REM sleep. Significant interactions (group x sleep/wake or group x sleep stage) were further explored using paired t-tests comparing mean values in hypertensives and normotensives for the different sleep or wake stages. For post-hoc testing, Fishers's least significant difference (LSD) correction was applied to account for increased error probability due to multiple comparisons. A *P*-value <0.05 was considered significant.

## Results

### Characteristics of sleep

Nocturnal sleep in hypertensive men did not differ from sleep in the normotensive group (all *P*>0.1; Table 2).

**Table 2:** Sleep characteristics in normotensive or hypertensive subjects (mean±s.d.).*

	Normotensives			Hypertensives		
Total Sleep Time (min)	398.5	±	30.1	397.3	±	24.1
Sleep Stages						
W %)	5.9	±	5.6	9.6	±	8.6
S1(%)	12.4	±	8.1	10.6	±	5.7
S2 (%)	44.8	±	11.6	43.8	±	9.4
SWS (%)	16.1	±	4.4	18.6	±	5.3
REM (%)	20.7	±	4.8	17.4	±	4.6
Sleep onset latency (min)	23.0	±	30.1	23.7	±	24.1
S2 latency (min)	5.9	±	2.5	6.0	±	5.0
SWS latency (min)	28.6	±	25.9	23.6	±	23.6
REM latency (min)	70.0	±	18.9	108.8	±	50.2

*There were no significant differences in sleep parameters between normotensive and hypertensive subjects.

### Effects of Sleep on Catecholamine Concentrations

**Figure 1:**
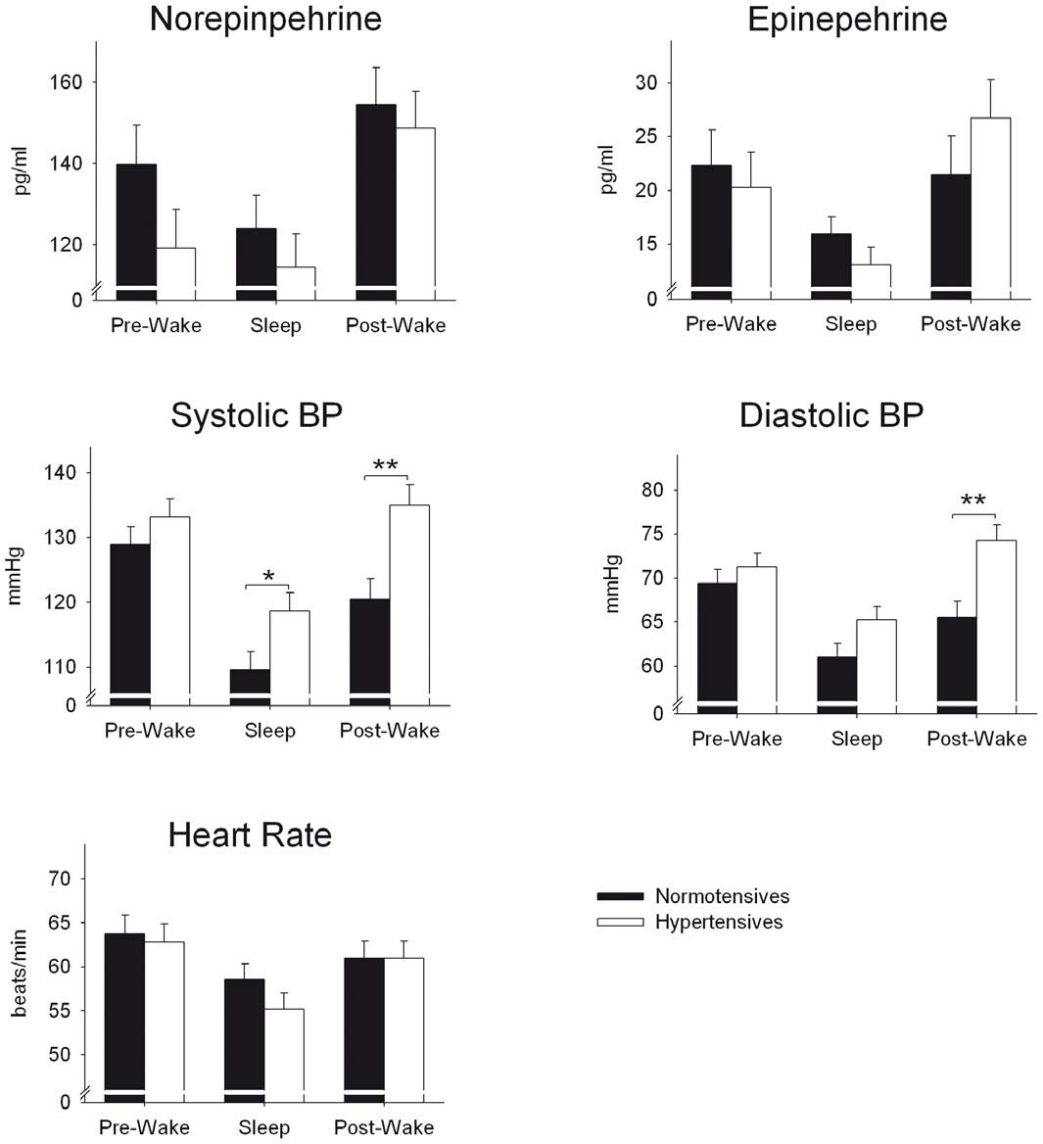
Means ± S.E.M. Norepinephrine and Epinephrine plasma concentrations, blood pressure (BP) and heart rate during 3-h periods of wakefulness prior to (pre-wake) and after sleep (post-wake), and during the 7-h sleep period for hypertensives (white) and healthy normotensive controls. *P<0.05; **P<0.01, for pairwise comparisons between hyper- and normotensives.

**Figure 2:**
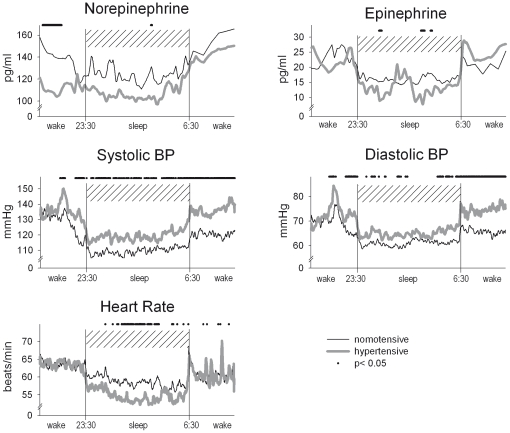
Heart rate, blood pressure and plasma catecholamines in 12 normotensive (thin line) and 12 hypertensive men (thick line) during nocturnal sleep and a period of wakefulness of 3.5 hours before and after the sleep phase. Heart rate and blood pressure were measured continuously, blood for the determination of catecholamines was drawn every 10 min during the sleep period. Profiles were averaged across individuals and time locked to sleep onset and awakening in the morning, respectively. Bars indicate significant differences (*P*<0.05) for post hoc comparison when ANOVA indicated overall significance for the factor condition (night-time vs. daytime sleep).

In contrast to the notion of an sympatho-adrenergic over activation in hypertension, we did not observe any significant group differences between *average* levels of NE or E during the pre-wake, sleep and post-wake periods, respectively (main effect group NE: F_(1,22)_  = 1.1, E: F_(1,22)_  = 0.0; both *P*>0.30). Generally, the concentrations of NE and E were significantly lower during sleep than during wakefulness (main effect sleep/wake NE: F_(2,44)_  = 23.5, p<0.001; E: F_(2,44)_  = 6.1, *P*<0.01; Figure 1), and, these general effects were similarly observed in hypertensives as well as normotensives as indicated by non-significant interaction terms (interaction group * sleep/wake for NE and E concentrations: both *P*>0.28). In an analysis of all succeeding time points of blood sampling during the periods of interest (Figure 2), NE concentrations were revealed to be even significantly lower in the hypertensives as compared to the normotensive controls during most of the pre-sleep wake interval. During sleep, hypertensives still exibited lower NE levels on a descriptive level, although these differences were non-significant (see Figure 2). During sleep, plasma catecholamine concentrations distinctly varied depending on the sleep stage, with linearly decreasing values as sleep deepens, reaching minimal levels during REM sleep (main effect of stage for NE and E concentrations, respectively: F_(4,88)_  = 4.95; *P*<0.01 and F_(4,88)_  = 7.36; *P*<0.001). The effect of sleep stages on NE and E concentration did not differ between hypertensives vs. normotensives (interaction group * sleep stage: F_(4,88)_  = 2.12; *P*>0.10 and F_(4,88)_  = 0.45; *P*>0.50).

To further evaluate our non-significant findings, we conducted post hoc power analyses using the software G*Power [18]. With regard to the comparison of average NE and E levels between hyper- and normotensives, we can safely exclude large effect sizes (f≥0.6 or d≥1.2) with a probability of 1-β>80%. However, we would like to emphasize that descriptively, the values for E and NE were even lower in early hypertensives as compared to normotensives. Thus, the descriptive effect was in the opposite direction (sometimes even significantly, see Figure 2). An increase in statistical power by increasing the number of participants would therefore detect a hypoadrenergic state rather than a hyperadrenergic state in our study. With regard to the interaction effects (group x sleep/wake and group x sleep stages, respectively), we can safely exclude small to medium effect sizes (f≥0.2 or d≥0.4) with a probability of 1-β>80%.

### Effects of Sleep on Cardiovascular Parameters

Overall, systolic and diastolic blood pressure during pre-wake, sleep and post-wake periods were significantly higher in hypertensive than normotensive participants (129±2.5/70.3±1.4 mmHg vs. 119.7±2.5/65.3±1.4 mmHg, F_(1,22)_ = 7.0 and 6.1, respectively, both *P*<0.03). In addition, blood pressure was generally lower during sleep as compared to wakefulness in both groups, indicating that in both groups some sleep-related blood pressure dipping evolved. However, the modulating influence of sleep vs. wakefulness on blood pressure differed between hypertensives and healthy controls: While the difference in systolic blood pressure between both groups was small during sleep (118.7±3.5 mmHg vs. 109.6±1.9 mmHg, *P*<0.04) and non-significant during the wake period before sleep (pre-wake period: 133.2±3.2 mmHg vs. 129.0±2.8 mmHg, *P*>0.28), hypertensives demonstrated highly significant increases in systolic blood pressure during wakefulness after sleep (135.0±3.3 mmHg vs. 120.5±3.0 mmHg, *P*<0.005; interaction group * sleep/wake: F_(2,44)_  = 3.7, *P*<0.04, Figure 1). A similar pattern was observed for diastolic blood pressure, with the highest differences between hypertensives and normotensives occurring in the morning after sleep (74.3±2.0 mmHg vs. 65.6 mmHg, *P*<0.004; interaction group * sleep/wake: F_(2,44)_  = 5.6, *P*<0.01).

Heart rate was also generally lower during sleep as compared to wakefulness (F_(2,44)_ = 27.2; *P*<0.001) in both groups. During sleep, heart rate tended to be lower in hypertensives (Figure 2), although the interaction between hyper- and normotensive participants did not reach significance (main effect and interaction: both *P*>0.16).

There was also an influence of the different sleep stages on systolic and diastolic blood pressure as well as on heart rate (F_(4,88)_  = 16.8, 13.8, and 27.4, respectively, all *P*<0.001). Consistent with our previous report [14], systolic and diastolic blood pressure as well as heart rate decreased with increasing depth of NonREM sleep and increased during REM sleep to levels comparable with those during intermittent wake periods. We did not observe any modulating influence of hypertension vs. normotension on this sleep-stage specific pattern of cardiovascular parameters (interaction group * sleep stage: all *P*>0.50).

### Relation between the morning increase in blood pressure and catecholamine concentrations

**Figure 3:**
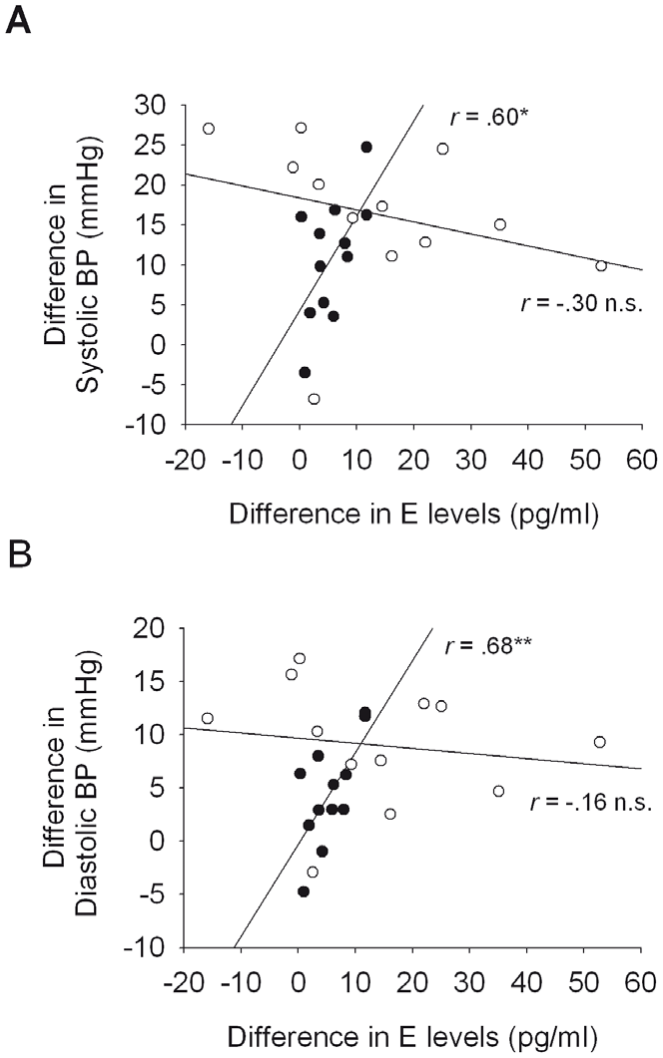
Correlation between the morning rise in epinephrine (E) levels and (A) systolic blood pressure as well as (B) diastolic blood pressure. The morning rise was defined as the absolute differences between average values during wakefulness after sleep minus average values during the sleep period. Normotensives (colsed circles) exhibited significant positive associations between the morning rise in E levels and blood pressure (BP), which was not the case in hypertensives (open circles). *P<0.05; **P = 0.01, for significance of correlation coefficients.

Because the largest difference between hypertensives and normotensives in blood pressure occurred in the morning after sleep, we further analyzed the relation between this increase and catecholamine concentrations. We defined the morning rise by the difference between the mean of the post-wake period and the mean of the preceding sleep period. As indicated by the above reported analyses, for systolic and diastolic blood pressure this morning rise was on average higher in the hypertensives than in the normotensives (Systolic: +16.3±2.7 mmHg vs. +10.9±2.2 mmHg; Diastolic: +9.0±1.6 mmHg vs. +4.5±1.4 mmHg; *P*<0.05), whereas the corresponding rise in E and NE levels did not significantly differ between the groups (NE: +34.2±5.0 pg/ml vs. +30.5±5.4 pg/ml; *P* = 0.61; E: +13.7±5.3 pg/ml vs. +5.5±1.1 pg/ml; *P* = 0.16). Correlation analyses in the normotensive group revealed, as expected (e.g., [14], [19]), that the morning rise in systolic and diastolic blood pressure was strongly correlated with the morning rise in E levels (*r* = 0.60 and 0.68, respectively, for systolic and diastolic blood pressure, both *P*<0.04, see Figure 3). Similarly positive but non-significant correlations were observed with the morning rise in NE values (systolic BP: *r* = 0.55, *P* = 0.07; diastolic: *r* = 0.38, *P* = 0.23). In striking contrast, hypertensives exhibited no positive correlation between the morning rise in blood pressure and catecholamine levels, neither for E (*r* = −0.30 and −0.16; both *P*>0.35; see Figure 3) nor for NE (*r* = −0.14 and −0.04; both *P*>0.60). The correlation coefficients for both wake-associated surges in systolic and diastolic blood pressure with E (both *P*<0.04) differed significantly between normotensives and hypertensives.

## Discussion

The main result of the present study is that young participants with mild newly diagnosed hypertension did not exhibit any strong increase in NE and E levels during nocturnal sleep or during preceding and succeeding 3-hour periods of wakefulness, in spite of highly reliable differences in blood pressure. On a descriptive level, hypertensives demonstrate even lower NE levels during wakefulness before sleep as well as during sleep, although these differences were not significant. During wakefulness in the morning after sleep, E and NE levels were almost identical between both groups, although increases in blood pressure from sleep to wakefulness were distinctly stronger in the hypertensives. Furthermore, whereas the morning increase in blood pressure occurred in parallel with an increase in catecholamine levels in normotensives, these parameters were completely unrelated in hypertensives.

The lack of a strong increase in catecholamine concentrations in our hypertensive participants stands in contrast with the common view that sympathetic overdrive is responsible for the hypertensive state and actively participates in its early development [1], [4]. Several studies have reported increased plasma catecholamine levels in hypertensive patients (see [1], [20], for reviews), and direct recordings from sympathetic nerve traffic to the vasculature have revealed potentiated responses in hypertensive patients, suggesting that increased NE levels result from an enhanced vasoconstrictive sympathetic drive [3], [21]. This and further evidence have led to the assumption that hypertension is a “hyperadrenergic state” [1], [22]. However, those studies were almost exclusively performed in waking subjects who often may not have been in a strictly horizontal position. In the present study care was taken that subjects were entirely relaxed an remained in a completely supine position throughout the experimental period, leaving the possibility that the adrenergic overactivation seen in hypertensive patients in previous studies depends on some additional orthostatic or stress-related stimulation [19].

In contrast to the catecholamine levels, blood pressure was as expected elevated in our hypertensive participants compared to the normotensive controls throughout sleep and wakefulness. Importantly, blood pressure elevation in the hypertensives was most pronounced during morning wakefulness after sleep, and this elevation was not paralleled by any similar elevation of catecholamine levels in the hypertensive participants. Moreover, whereas in the normotensive subjects the morning increase in blood pressure was positively correlated with the increase in catecholamine levels, mainly in epinephrine levels, no such correlation was found in the hypertensive subjects. These results open the possibility that sleep-related blood pressure regulation is dysfunctional in early hypertension, with sympatho-adrenergic signaling exerting a diminished control over blood pressure. The particular elevation of blood pressure in hypertensives following morning awakening being rather unrelated to concurrent adrenergic activity, might origin from a disturbance in sleep mechanisms specific to the control of blood pressure, rather than from impaired sleep or sleep stages as a whole, since sleep architecture in our hypertensive subjects was similar to that in normotensive controls. Evidence for the notion of an active control of waking blood pressure levels during previous sleep has indeed been provided by a recent study that pharmacologically prevented the reduction (‘dipping’) in blood pressure during sleep by a continuous infusion of phenylephrine, an alpha-adrenoceptor agonist that does not pass the blood– brain barrier [5]. The infusion significantly increased blood pressure during sleep, with only minimal effects on sleep architecture and quality. The disturbance of sleep-related dipping induced a strong counter-regulation with a shift of the baroreflex threshold towards lower blood pressure levels, which sustained for several hours during the subsequent waking period. In our hypertensive subjects, reduced dipping of blood pressure during sleep could have likewise contributed to an exaggerated increase in blood pressure during subsequent morning wakefulness. The present data add to those previous findings in suggesting that, at least in early stages of hypertension, blood pressure counter-regulation upon awakening involve signals other than sympatho-adrenal activation.
